# High performance recycled CFRP composites based on reused carbon fabrics through sustainable mild solvolysis route

**DOI:** 10.1038/s41598-022-09932-0

**Published:** 2022-04-08

**Authors:** W. Ballout, N. Sallem-Idrissi, M. Sclavons, C. Doneux, C. Bailly, T. Pardoen, P. Van Velthem

**Affiliations:** 1 Institute of Condensed Matter and Nanosciences - Bio & Soft Matter (IMCN/BSMA), UCLouvain, 1 Place Croix du Sud, box: L7.04.02, 1348 Louvain-la-Neuve, Belgium; 2 Institute of Mechanics, Materials and Civil Engineering, UCLouvain, Place Sainte Barbe 2, 1348 Louvain-La-Neuve, Belgium

**Keywords:** Chemistry, Engineering, Materials science

## Abstract

A novel environmentally friendly recycling method is developed for large carbon-fibers reinforced-polymers composite panels whose efficiency is demonstrated through a proof-of-concept fabrication of a new composite part based on recycled fibers. The recycling process relies on formic acid as separation reagent at room temperature and atmospheric pressure with efficient recycling potential of the separating agent. Electron microscopy and thermal analysis indicate that the recycled fibers are covered by a thin layer of about 10wt.% of residual resin, alternating with few small particles, as compared to the smooth virgin fibers. The recycled composites show promising shear strength and compression after impact strength, with up to 93% retention of performance depending on the property as compared to the reference. The recycled carbon fibers can thus be reused for structural applications requiring moderate to high performances. The loss of properties is attributed to a lower adhesion between fresh epoxy resin and recycled carbon fibers due to the absence of sizing, partly compensated by a good interface between fresh and residual cured epoxy thanks to mechanical anchoring as well as chemical reactions. The room temperature and atmospheric pressure operating conditions combined to the recyclability of the forming acid contribute to the sustainability of the entire approach.

## Introduction

Despite the high economical and environmental processing cost of carbon fibers reinforced polymer matrix composite (CFRP) solutions, the global demand for these materials keep raising due to the increasing number of applications in cars, boats, trains, windmills and airplanes. The main advantages stem from the high strength and stiffness over density performance indicators. The superior properties come from the carbon fibers (CFs) while the matrix, such as epoxy, ensures efficient load transfer provided that good adhesion with the fibers and sufficient ductility are sufficient to avoid premature failure.

However, CFRPs present challenges of environmental nature related first to the high embodied energy associated to CFs production and of the polymer-based matrices^1^. Furthermore, large volumes of waste are created at the end of the operational life time. Recycling is the obvious solution to decrease the energy impact over the life cycle. The existing end-of-life solutions have indeed limitations: incineration offers poor energy efficiency and generates polluting emissions while mechanical recycling only recovers lower performance reinforcements. Furthermore, landfill, while preferable as compared to incineration, will be restricted in a near future by legislation pushed by directives adopted by European Union to promote recycling and the use of waste as a resource^1–4^.

New attempts have been made to recover individual CFs from composites waste by developing different physico-chemical approaches. The furthest advances have focused on pyrolysis and solvolysis treatments^5–7^. However, these processes often require prior milling or grinding steps, intense energy consumptions and often the use of environmentally unfriendly solvents. The result is often: (i) a product with insufficient quality which can only be re-introduced in the market for non-critical applications due to fiber length shortening, very drastic conditions and intrinsic mechanical properties abatement of the CFs^7,8^ and (ii) an organic fraction recovered from the resin but, due to its very complex mixture varying from one composite to another, only exploited for energy recovery^9^. Recently Wang et al. proposed a strategy to recycle valuable oligomers from cured epoxy resin composites via a selective cleavage of tertiary carbon–nitrogen bond using acetic acid associated to AlCl_3_ under mild conditions^10^. The claim was that the oligomers of the cured epoxy can be preserved, recycled and reused in resin manufacturing. The resulted CFs are individualized and, when tested, exhibit almost their original tensile strength and elastic modulus.

The development of recycling processes preserving the fiber woven architecture of cured waste remains a bold challenge. Indeed, unsized CFs are much more difficult to manipulate in remanufacturing and require realignment operations^9,11^. Novel technologies adressing this possibility have emerged only recently. For instance, Yu et al. have proposed a lab scale technique for recycling CFs from special composites with almost 100% yield^8^. The matrix (fatty acid mixture and epoxy) is a covalent adaptative network which is capable of transesterification type bond exchange reactions, fully dissolving in ethylene glycols by soaking at 180 °C. The resulting dissolved polymer, after solvent elimination, when repolymerized, leads to the same thermomechanical properties as the fresh polymer. The woven fibers were reused in new applications with similar mechanical pattern and dimensions^8^.

In this same context of recovered woven fibers, sub-critical and supercritical fluids such as water or alcohols offer new opportunities to recycle multi-layered composites. The recycled woven fabric layers, with retention of the fiber architecture, could be directly reused after minimal further processing to fabricate fiber composites. The residual recycled resin could be incorporated into fresh resin and cured. However, the equipment is quite demanding since it must be capable of operating safely at the necessary conditions for supercritical fluid processing (high pressure and temperature). A catalyst is often necessary^12^, but not always^13,14^. In this last case, a semi-flow type reactor allows the loading of larger multi-layers (200 mm × 45 mm × 2 mm). The recovered CFs maintained the shape of the plain fabric, at least in light of the published pictures, at the center of the recovered woven^15^. The studied sample could be mechanically constrained in order prevent possible entanglement with the reactor stirrer^12^. Studies revealed that efficient temperature and pressure levels significantly depend on the type of epoxy resin^9^. Unfortunately, the reaction rate when using larger pieces of composites upon scale up could be limited by diffusion requiring a new optimization of the selected parameters^9^.

Limited effort has been directed toward acid digestion as these processes are rightly considered dangerous in terms of health and environmental impact. Nitric acid has been successfully used to recycle CFs^16^. Other teams have also succeeded in recovering CFs of high quality using sulphuric or acetic acid as pretreatment and a solution of hydrogen peroxide for the oxidative degradation of the epoxy resin^17,18^. However, in these studies, the recovered CFs are too small, as the composite had to be cut into small slices (about 10 mm), and the woven fabric shape structure is not preserved.

This paper presents the main findings of an investigation on the reuse, relying on formic acid digestion, of fully reclaimed intact woven CF fabrics originating from an aerospace-grade composite made with a high-performance epoxy resin used in primary structural applications. The recycled CF mat in original A3 size was processed with the same epoxy resin and the same conditions (resin transfer molding) as the virgin composite. The procedure used in this study to extract the woven fabric from composite parts relies on the same principle as an existing wet industrial process set to separate aluminum-polyethylene composite packaging materials^19^. The process uses formic acid as separation reagent. The choice of formic acid has been motivated by several arguments. Among organic acids, formic acid presents the advantage that it is the strongest one with the highest acid density and the lowest chemical oxygen demand. Moreover, compared to inorganic acids, formic acid does not release nitrogen, phosphorous or sulfur that can cause eutrophication. Environmentally speaking, formic acid is an efficient and environmentally compatible alternative. In the literature, several routes have been investigated in order to sustainably recycle formic acid^20–25^. Moreover, nowadays, the main challenge of society is to combat the global warming, in particular due to CO_2_ emissions. In this sense, Carbon Capture and Storage (CCS) and Carbon Capture Utilization (CCU) techniques have been acknowledged as an important research and development priorities of the European Energy Union, to reach 2050 climate objectives in a cost-effective way. One solution to capture CO_2_ is to use formic acid^20,25^. As an example, Zhao et al. demonstrated, via a proof of concept, that formic acid can be reformed by using a bio-catalytic system based on CO_2_ capture^20^. The tests were carried out at room temperature, under atmospheric pressure and in a static mode to eliminate the cured epoxy resin and to regenerate the starting structured woven fabric.

Therefore, the present contribution carries two complementary objectives:

1. To assess the effect of the recycling process on the degradation of the reinforcement potential of the carbon fibers. Scanning electron microscope (SEM) and thermo-gravimetric analysis (TGA) were used to characterize the residues of epoxy on the surface of the recovered carbon fibers.
2. To investigate the effect of the fiber recycling on the composite performances. The microstructure arrangement of the resulting panel after acid treatment was characterized by SEM and the local properties by nanoindentation. Moreover, several mechanical properties of the composite specimens made out of virgin and recycled fibers having identical stacking sequence have been determined including inter-laminar shear strength (ILSS), compression, compression after impact (CAI) and Iosipescu shear tests.

## Materials and experimental details

### Materials

The epoxy resin used in this study is the HexFlow® RTM6 supplied by Hexcel Composites, which is qualified for aerospace applications. The carbon fiber (CF) fabrics used as reinforcement consist of HexForce® G0926 (HTA 6k) with a 5 harness satin weave (375 g/m^2^) manufactured by Hexcel Composites. The solvent for the acid digestion is the formic acid 98–100% from Sigma-Aldrich.

### Fabrication of reference and recycled composite

A reference composite panel based on the RTM6 epoxy resin and neat carbon fabrics was manufactured using the vacuum-assisted resin transfer molding process (VARTM) from Isojet. After complete impregnation at 100 ml/min, a dwell pressure of 6.5 bars was applied for 2 h to minimize porosity. The RTM panel comprised eight layers of carbon fabrics of 420 mm × 300 mm dimensions with an isotropic [0/90]_4_ lay-up and a nominal thickness of about 3.5 mm. The curing cycle consists of a 1.5 °C/min ramp from 80 to 180 °C, followed by a 2 h isothermal step. Another composite panel, made in the same conditions, was produced and immerged in a formic acid bath for 48 h. The acid digestion was carried out under atmospheric pressure and at room temperature without any stirring (static mode). The eight recovered CF layers were taken out of the bath, rinsed with distilled water and finally dried under vacuum at 70 °C for 24 h. The recovered carbon fabrics were placed in the RTM mold cavity and subjected to a new impregnation with fresh RTM6 epoxy resin. The latest composite panel is called “recycled composite” and compared to the reference one.

**Figure 1:**
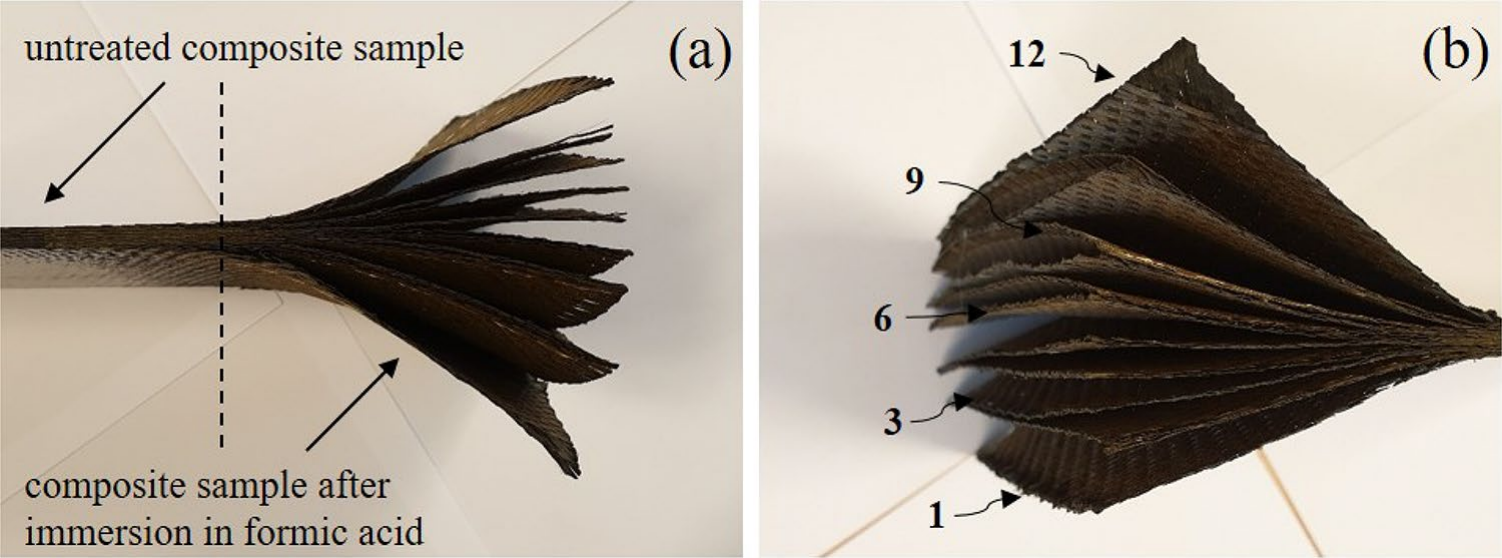
Illustration of separated CF layers after partial immersion in formic acid.

Figure 1 illustrates an example of a composite made of twelve carbon layers, partially immersed in formic acid. Figure 1a shows both untreated and treated parts of the same composite panel while Fig. 1b shows the twelves well-separated CF plies.

### Characterization methods

#### Scanning electron microscopy (SEM)

Specimens for SEM analysis were mounted on stubs and coated with 8 nm chromium layer (Cressington sputter 208HR) to produce a thin conductive layer, minimizing degradation and drift due to thermal expansion. SEM analyses were performed on polished surfaces in a Jeol FEG SEM 7600F operating at 15 keV with a working distance of 8 mm.

#### Thermogravimetric analysis (TGA)

Thermo-gravimetric measurements were performed using a TGA/SDTA851e from Mettler Toledo to determine the amount of residual epoxy resin still present on the treated fiber after wet treatment. Tests were performed under air environment with a flow rate of 50 ml/min. Samples of around 15 mg are heated at 10 °C/min. The evolution of the sample weight was recorded from 25 to 900 °C. Reported values were average over a minimum of 3 tests.

#### Fourier transform infrared spectroscopy (FTIR)

FTIR was carried out by a Nicolet™ iN™ 10 infrared microscope from Thermo Fisher Scientific in attenuated total reflectance mode (ATR). The FTIR absorption spectrum of recycled carbon fibers was recorded in the wavenumber range from 4000 to 400 cm^−1^ with a spectral resolution of 4 cm^−1^.

#### Nanoindentation

Nanoindentation tests on the reference and recycled composites were performed with a Agilent G200 equipment relying on the Dynamic Contact Module (DCM), which allows for accurate measurements at low loads and indentation depths. The Continuous Stiffness Measurement (CSM) mode was used to obtain a continuous measure of the hardness and the modulus during the test. The CSM frequency was set up to 75 Hz and the maximum indentation depth on both samples was 500 nm. The target strain rate was set at 0.05 s^−1^. The indents were made using a Berkovich tip inside the resin region in regions of high fiber content oriented perpendicularly to the top surface. The goal is to investigate any difference of matrix behaviour between the reference and recycled composite at locations very near the fiber interfaces. The regions are indeed the important one to ensure the load transfer to the fibers, possibly influence by the residues and presence/absence of sizing. In total, 30 indents per sample were made, to ensure statistical relevance.

The hardness is dtermined as: $H = \frac{{F_{max} }}{A}$, where *A* is the projected contact area. As the real contact area may differ from the apparent contact area due to sink-in or pile-up, we use the Oliver and Pharr method to estimate the contact area for a Berkovich tip, which gives: $A\left( {h_{c} } \right) = 24.65 h_{c}$, with *h*_*c*_ the contact depth. The magnitude of *h*_*c*_ is obtained based on Sneddon’s equation: $h_{c} = h_{max} - \frac{{\varepsilon F_{max} }}{S}$, where *S* is the contact stiffness, equal to the slope of the unloading.

The modulus *E* is determined from the reduced modulus *E*_*r*_ by: $\frac{1}{{E_{r} }} = \frac{{1 - \nu^{2} }}{E} + \frac{{1 - \nu_{i}^{2} }}{{E_{i} }}$ where *E*_*i*_ and *ν*_*i*_ are the properties of the indenter and $E_{r} = \sqrt {\frac{\pi }{A}} \frac{S}{2}$.

#### Interlaminar shear strength (ILSS)

The interlaminar shear strength tests were performed according to the EN 2563 standard. Rectangular specimens of 30 × 10 mm were tested with a rigid 3 points bending fixture on a Zwick Z250 universal testing machine operating at room temperature under a constant crosshead displacement speed of 1 mm/min. The span between the support cylinders was equal to 20 mm.

#### Compression

Compression tests were performed according to the ASTM D-6641 standard. Rectangular specimens of 140 × 12 mm were tested on a Zwick Z250 universal testing machine equipped with a 250 kN load cell. The specimen was clamped into the appropriate Wyoming compression test fixture (WTF) with 3 Nm torque. The crosshead speed was equal to 1.3 mm/min. A strain gage (EA-06-125EP-350 by Vishay) was glued on each side of the specimen with M-Bond 200 adhesive system (Vishay). The compression modulus is calculated in the strain range between 1000 and 3000 µStr and rejected if the bending at 2000 µStr is superior to 10%. Accordingly, the failure stress was rejected if the bending at failure was superior to 10%.

#### Compression after impact (CAI)

Compression after impact specimens were cut to 100 × 150 mm and subjected to a transverse impact of 24 J using an Instron Dynatup 9250HV impactor according to the AITM1-0010 standard. The size of the internal damage zone was determined by ultrasonic C-scan inspection. The damaged specimens were then loaded in a Zwick Z250 universal testing machine equipped with a load cell of 250 kN and tested using an in-plane compression fixture (WTF) in order to determine the residual strength.

#### Iosipescu shear test

The Iosipescu shear test consists of a V-notched specimen loaded according to ASTM D 5379. One side of the fixture is displaced vertically while the other side remains fixed and opposing force couples prevent any in-plane bending of the specimen. If properly executed, the stress state between the notches is a pure shear state, uniform in the minimum cross-section. The square area within the minimum cross-section covered by the strain gages is referred to as the test region in this study. The measurements were performed under dry conditions.

## Results and discussion

### Virgin and recycled carbon fibers analysis

#### Fiber morphology

**Figure 2:**
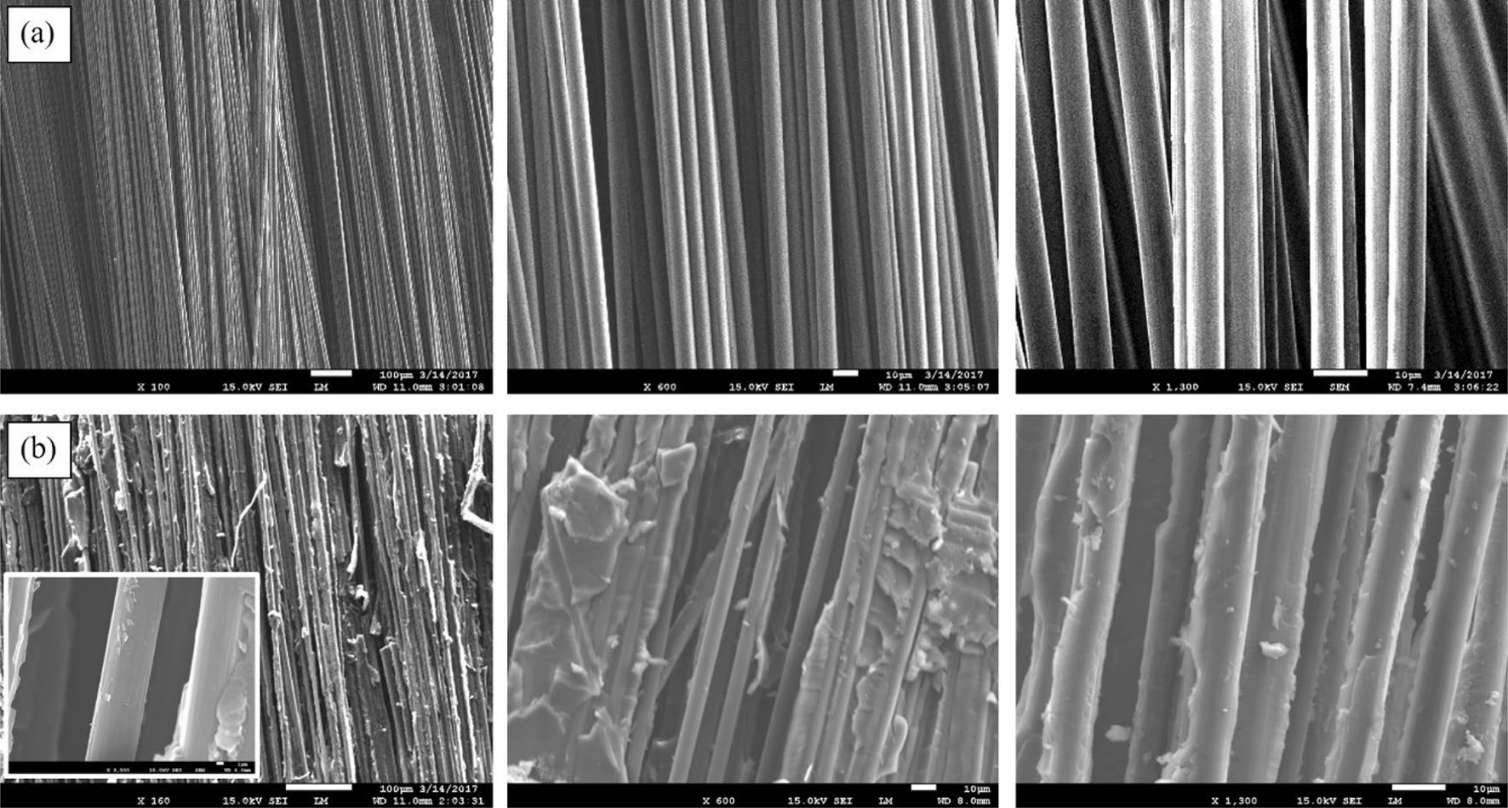
SEM images of (**a**) virgin and (**b**) recycled carbon fibers.

The morphology and surface quality of virgin and recycled carbon fibers are presented in Fig. 2. The “as received” CFs (Fig. 2a) show smooth fiber surfaces while the recycled carbon fibers (Fig. 2b) are covered by some residual epoxy resin layer, alternating with few small “particles” along the fiber’s axis. These particles result from an incomplete digestion of the cure resin (the exact amount of residual epoxy resin is quantified below by TGA). Indeed, epoxy residues are still present on the recycled carbon fibers showing that perfectly clean recycled fibers cannot be obtained even after 48 h in formic acid and at room temperature, although a fraction of the fibers are devoid of residual resin as illustrated in the insert of Fig. 2b. Interestingly, the applied chemical treatment does apparently not affect the quality of the surface of the carbon fibers, in particular the roughness.

**Figure 3:**
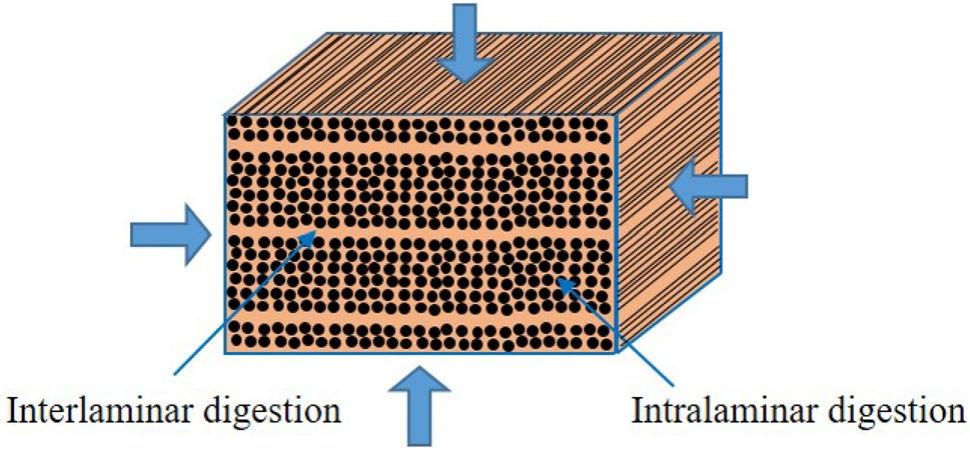
Schematic drawing of digestion in cross-section of composite.

Additionally, it seems that the digestion does not initiate everywhere at the same time nor at the same rate. Indeed, when a composite is immersed into formic acid, the preferential digestion or etching first takes place at the edges and at the interlaminar spots instead of the intralaminar level where the fiber density is much higher, making the acid digestion longer. A certain amount of residual epoxy is then left between the fibers (Fig. 2b). A schematic drawing of the digestion process in a cross-section of a composite is represented in Fig. 3.

#### Thermal stability characterization by TGA

**Figure 4:**
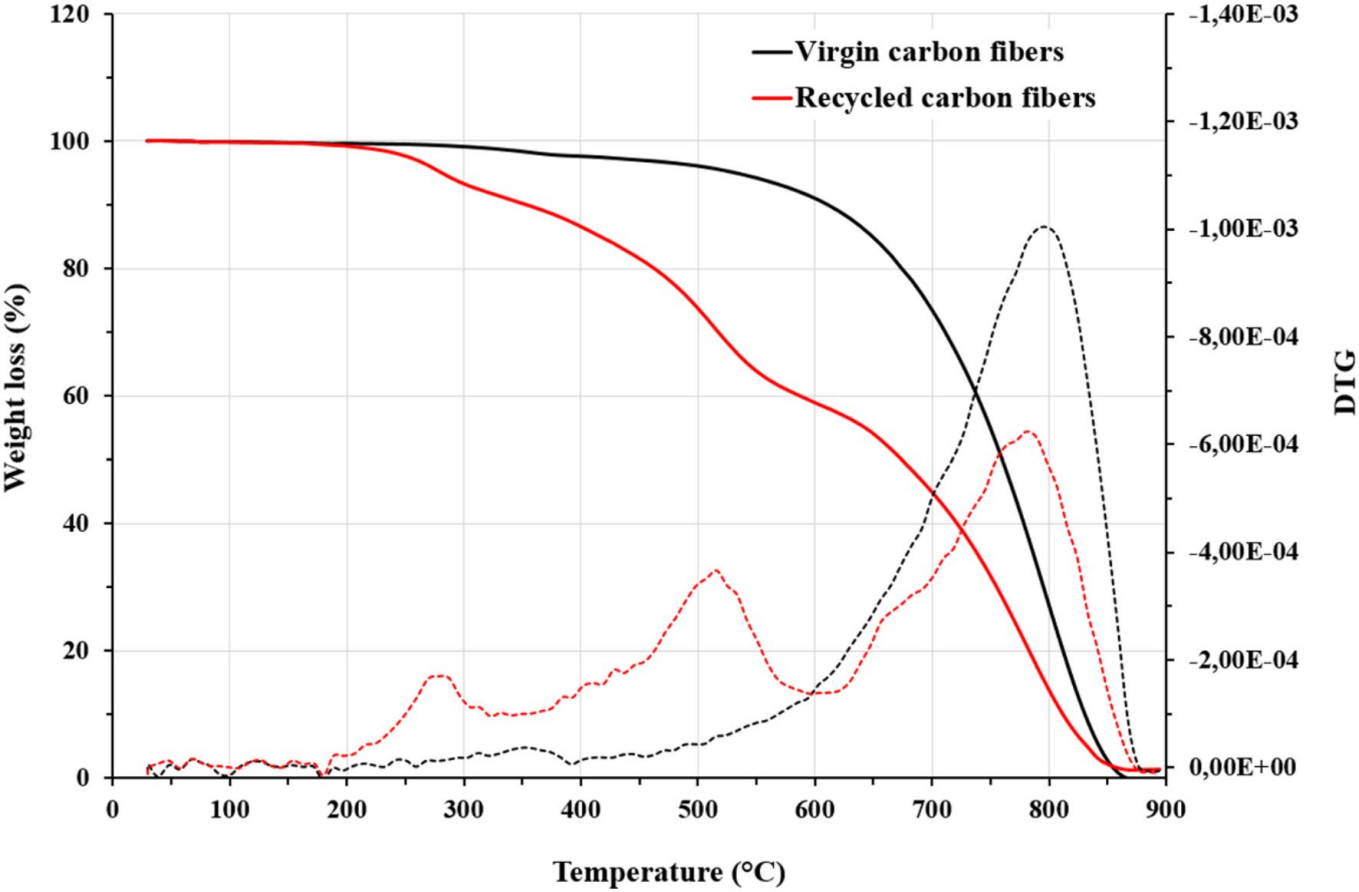
TGA and DTG curves of virgin and recycled carbon fibers.

The thermolysis behavior of virgin and recycled carbon fibers in air can be compared from their repsective mass loss as a function of temperature as shown in Fig. 4. The residual weights at 900 °C of the virgin and recycled CFs are close to 0 wt.% and less than 2 wt.%, respectively. The TG curve of the virgin CFs first shows a small weight loss of about 2 wt.% at temperatures between 200 and 400 °C which can be attributed to the fiber sizing decomposition. From 500 to 900 °C, the virgin fibers get progressively oxidized until total weight loss. In contrast, recycled CFs exhibit a more complex behavior with three oxidation peaks, which are ascribed to the thermal degradation of (i) the residual epoxy resin, (ii) the oxidation of the pyrolytic carbon and (iii) the oxidation of the carbon fibers, respectively as described by Yang et al.^26^. The weight loss corresponding to the decomposition of the sizing and to the thermal degradation of the residual cured epoxy resin is around 12 wt.% (determined at 400 °C) meaning that around 10 wt.% of epoxy resin remains on the recycled fibers after 48 h of treatment with formic acid at atmospheric pressure and room temperature.

### Characterization of the composite properties

#### Morphological evaluation

**Figure 5:**
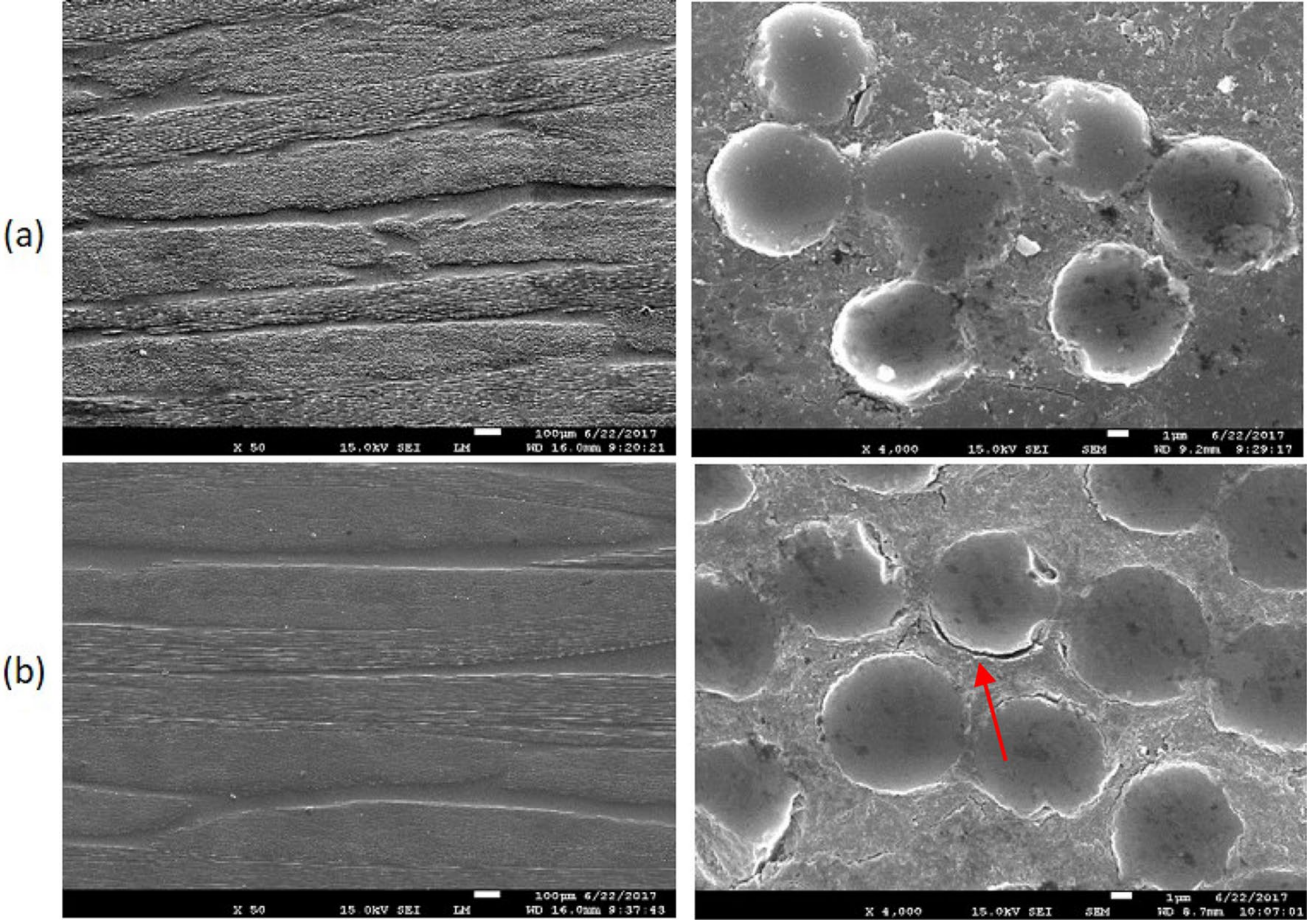
SEM images of cross-section of (**a**) virgin and (**b**) recycled composites.

The morphological characterization of the composite panels is investigated by SEM. Cross-section images of panels made of (a) virgin or (b) recycled carbon fibers with fresh epoxy resin are illustrated in Fig. 5. Although CFs are properly impregnated by the RTM6 epoxy resin, without any porosity in both cases, the presence of furrows (arrow on Fig. 5b) surrounding the recycled carbon fibers indicate a lack of adhesion between the recycled fibers and the epoxy matrix. The observed lack of adhesion can be attributed to the destruction of the fiber sizing by the digestion process.

#### Nanomechanical characterization of local properties

**Figure 6:**
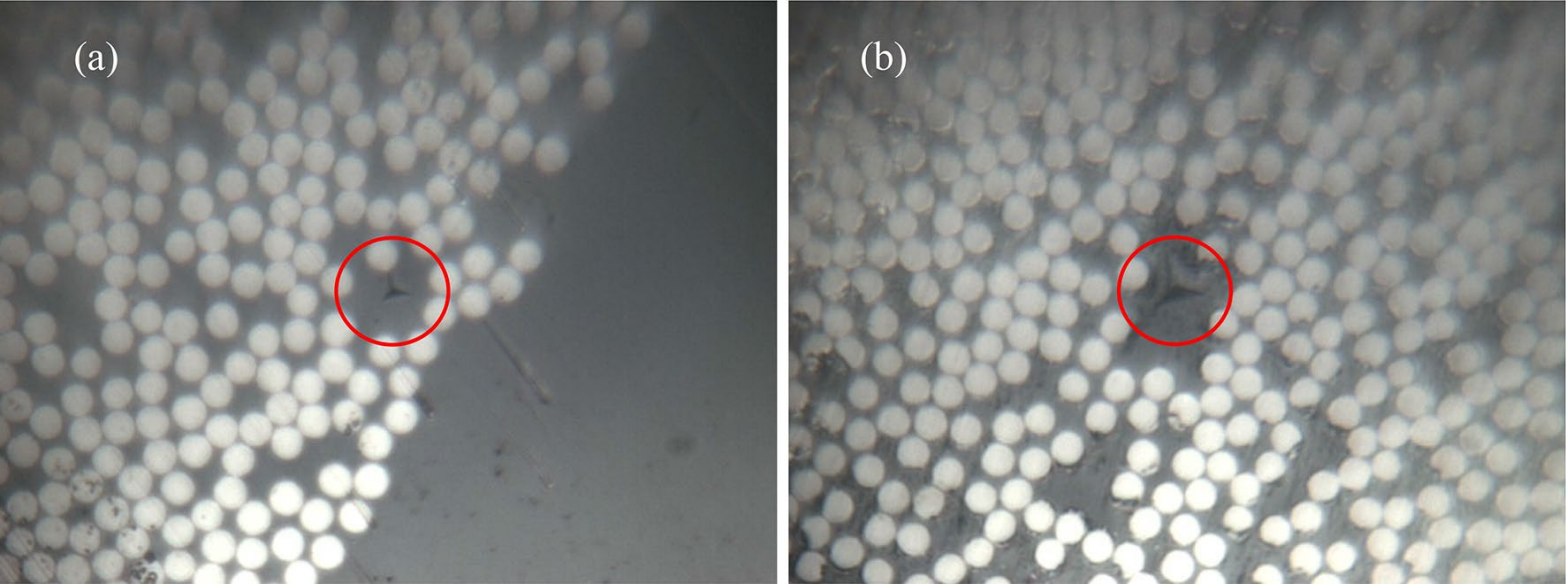
Optical images (40 × of magnification) of (**a**) reference and (**b**) recycled composite surfaces showing the indent point location (red circle).

Nanoindentation tests were performed to investigate the mechanical behaviour of the near fiber/matrix regions of the virgin (reference) and recycled composites. Figure 6 shows examples of indents performed very near the carbon fibers, sometimes as close as 1 µm, while avoiding significant mechanical interaction with the fiber on the elastic and plastic strains^27,28^.

**Figure 7:**
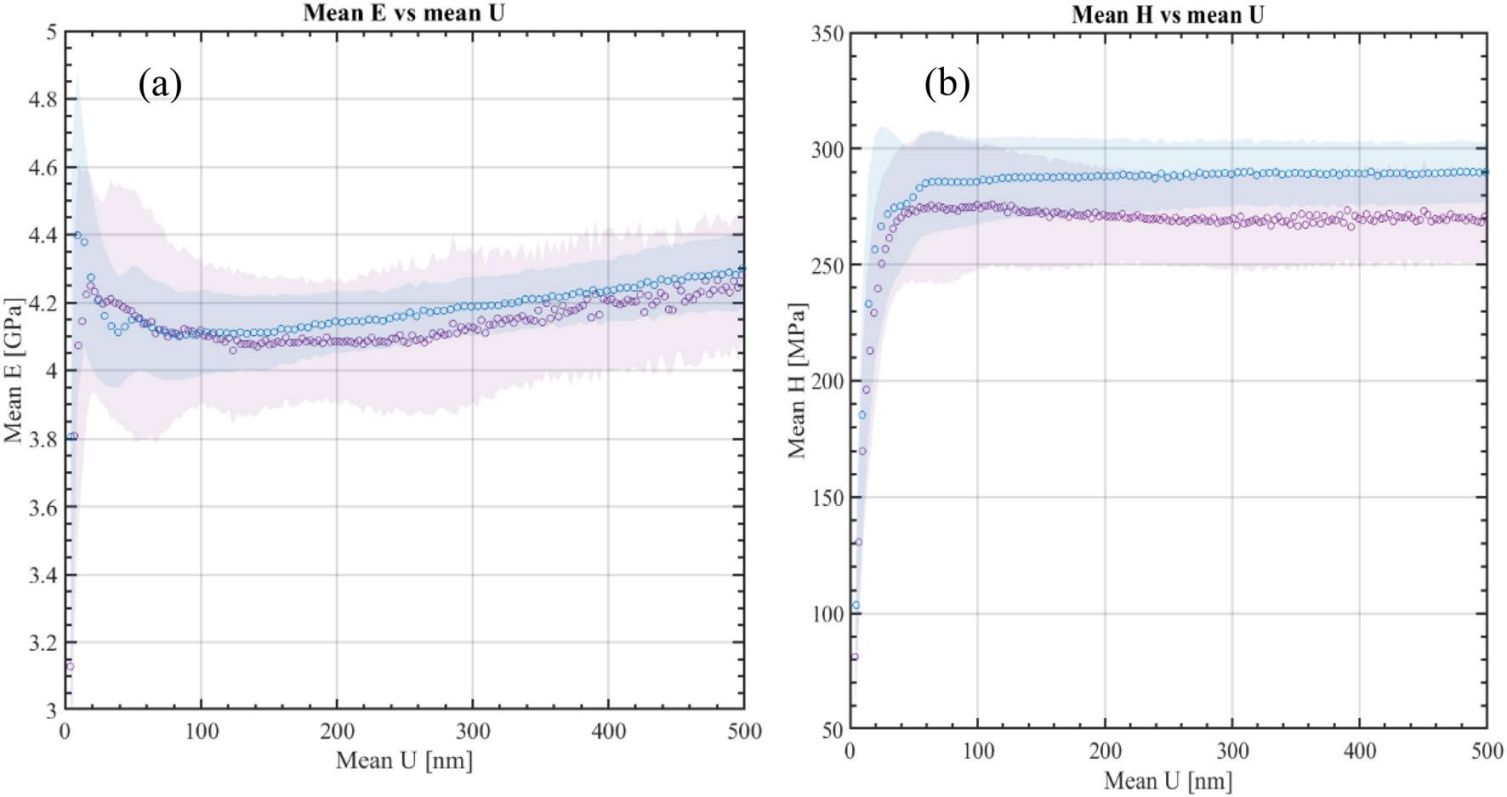
Reference versus recycled composites (**a**) moduli and (**b**) hardness as function of indent penetration depth.

Figure 7a describes the evolution of the modulus as a function of the penetration depth for reference and recycled composite samples. The result shows a similar behavior for both composites. However, the local hardness (Fig. 7b) of the recycled composite is lower by 7% as compared to the reference one. This means that the near fiber/matrix interface region is slightly affected by the conditions of curing of the recycled composite, in particular the presence of rsidues of epoxy resins.

#### Mechanical testing

##### Interlaminar shear strength (ILSS)

The ILSS values of the reference and recycled composite panels are determined to quantify the influence of the reclaimed carbon fibers on the resistance to delamination damage. The average maximum load and ILSS values of the virgin and the recycled composite panels are presented in Table 1.

**Table 1:** Maximum load and ILSS values of reference and recycled composite specimens.

Specimens	Maximum load (N)	ILSS (MPa)
Virgin composite (reference)	2790 ± 125	69.2 ± 2.4
Recycled composite	2810 ± 98	61.2 ± 1.5

The ILSS average value of the recycled composite is 12% lower compared to the reference composite. This result indicates that the ILSS property predominantly depends on the matrix with a minor contribution of the fiber-reinforcement. The presence of residual cured epoxy resin on the carbon fibers and the lack of sizing after formic acid treatment, as shown in Fig. 2b, explains the moderate decrease of the ILSS value.

##### Compression stiffness and strength

**Figure 8:**
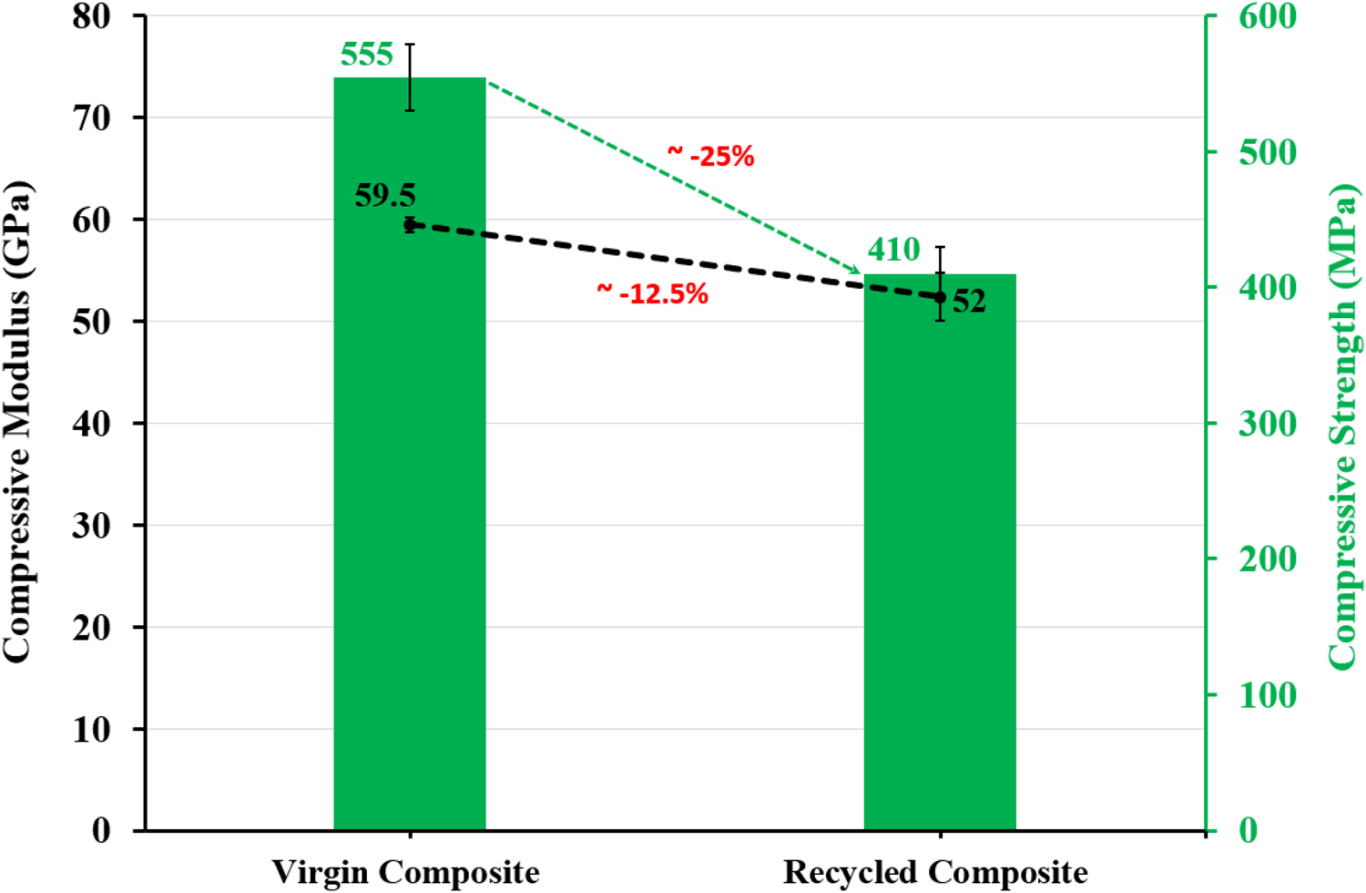
Compression modulus and compression strength of the virgin and recycled composites.

Figure 8 exhibits the compression modulus and compression strength of the virgin and recycled composites. A decrease by 12.5% and 25% is found for the recycled specimen compared to the reference, respectively. The strength is, as expected, more impacted than elastic stiffness. This decrease may be attributed, as discussed in the previous section, to the poor adhesion between the residual resin and the fresh resin, and between the unsized fibers and the fresh resin.

**Figure 9:**
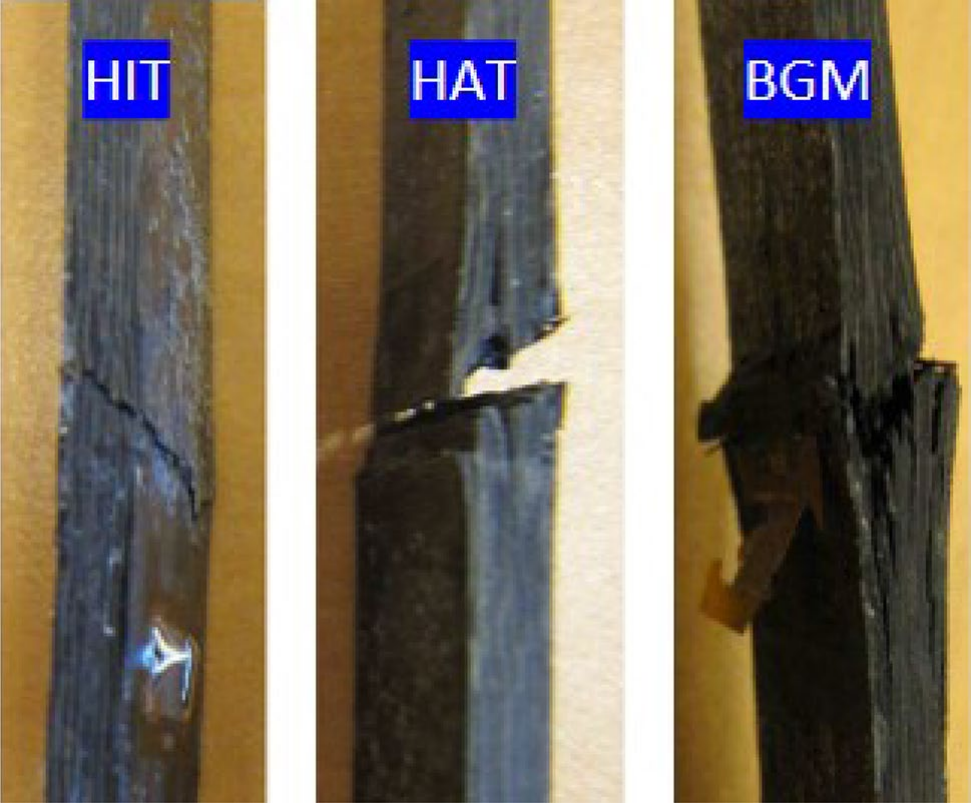
Failure modes observed in the recycled specimens: HIT failure mode, HAT failure mode and BGM failure one.

Examples of the different failure modes observed for the recycled specimens after loading in compression are illustrated in Fig. 9. A dozen of distinguishable failure modes is reported in the literature in the case of composite in-plane compression such as brooming in the middle of the gage length (BGM), crushing, delamination, Euler buckling, through-thickness, longitudinal-splitting, transverse shear, etc. The failure modes are identified here as BGM (brooming in gage length in the middle) and HAT (through the thickness at the grip at the top) which are considered as acceptable test according to ASTM D6641. The observed HIT mode (through the thickness in the grip at the top) is not acceptable in view of the test validity.

##### Compression after impact (CAI)

**Figure 10:**
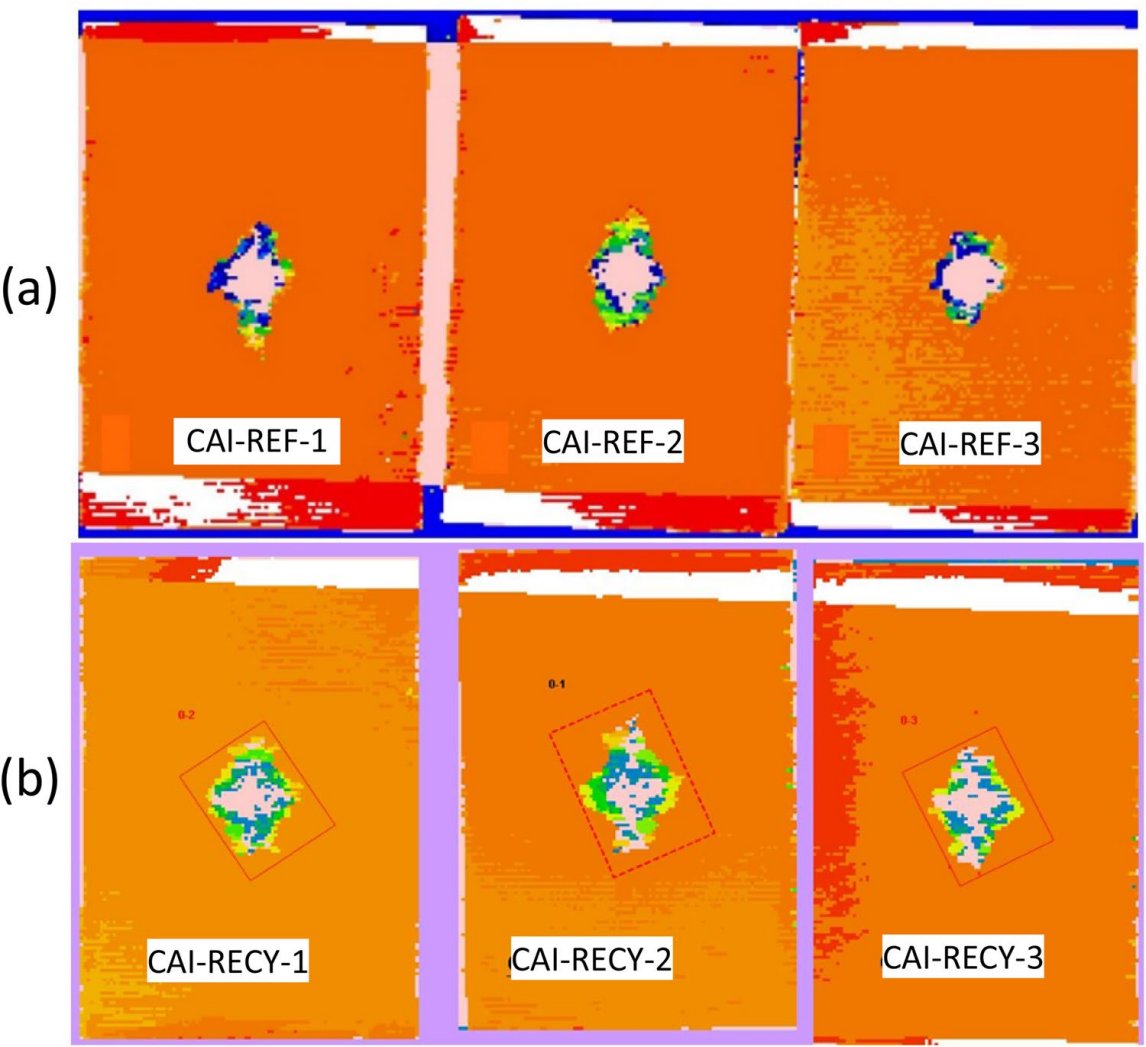
C-scan images of composites impacted at 24 J: (**a**) virgin and (**b**) recycled.

Virgin and recycled composites were impacted at static incident energy of 24 J. The delamination areas evaluated by C-scan measurements are shown in Fig. 10, with the quantitative values listed in Table 2. The shape of the delaminated area of both composites is elliptical with a larger area for the recycled case, corresponding to an increase of the delamination surface by 46% compared to the reference.

**Table 2:** Delaminated area, maximum force and CAI values of the virgin and recycled composites specimens.

Specimens	S (mm^2^)	F_max_ (N)	CAI strength (MPa)
Virgin composite	960 ± 160	63,400 ± 2180	220 ± 8.5
Recycled composite	1400 ± 233	51,600 ± 1820	145 ± 3.5

A comparison between the virgin and recycled composites in terms of maximum force and compression strength after impact is also given in Table 2. The recycled composite shows a decrease of the residual compression strength after impact by 33%. Impact properties are mainly dominated by the fibers, with a weak adhesion between the fibers and the matrix playing thus a major role.

##### Iosipescu shear test

**Figure 11:**
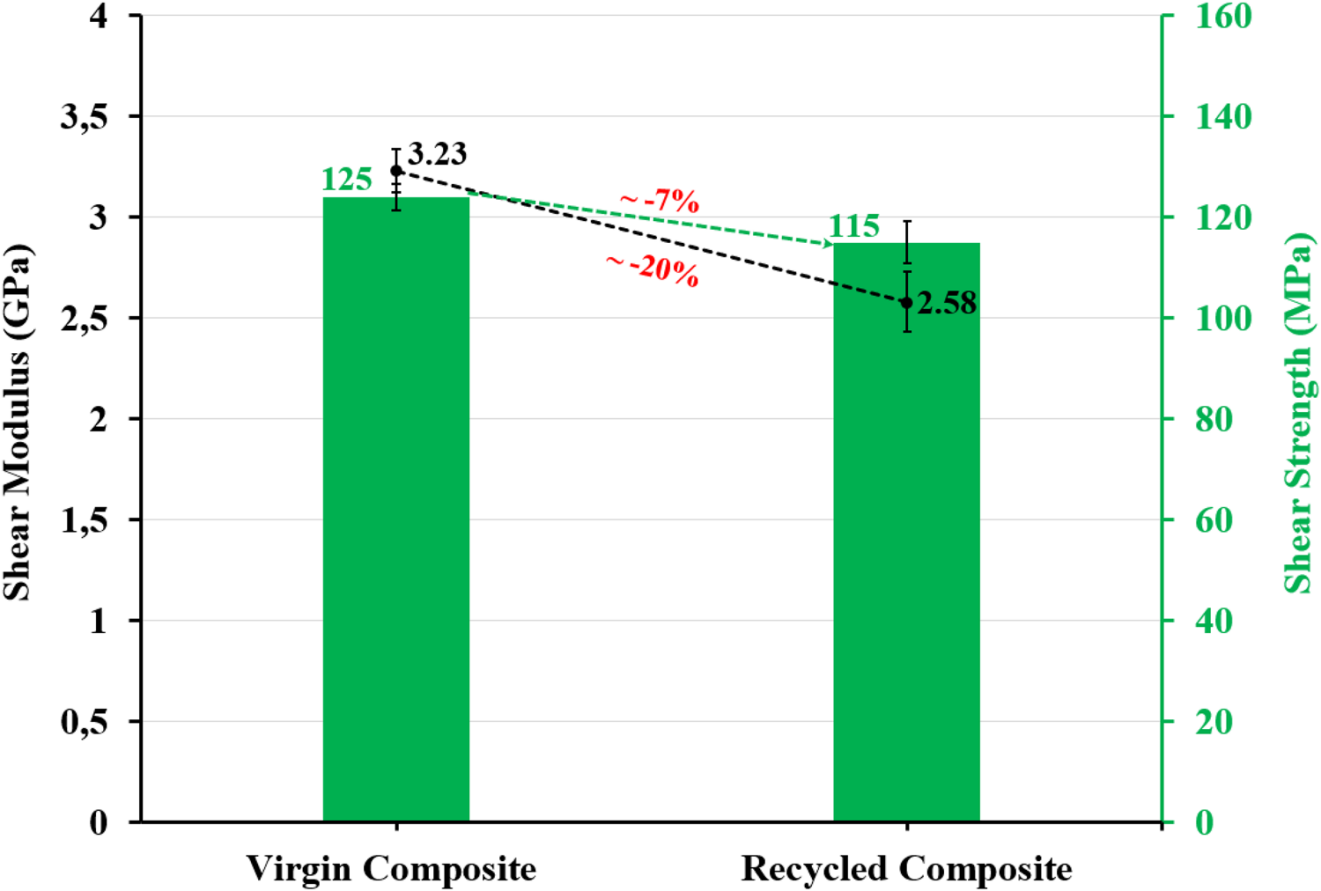
Shear strength and shear modulus evaluations of virgin and recycled composite specimens.

Fig. 11 presents the results for the shear strength (τ_12_) and shear modulus (G_12_) of virgin and recycled composites, respectively. A minimum of six specimens were tested for each composite material condition (reference or recycled). The recycled composite shows a 7% lower shear strength (115 MPa) than the virgin composite (125 MPa). The presence of recycled fibers reduces the shear modulus by about 20% compared to the reference.

### Overall view on the performances

**Figure 12:**
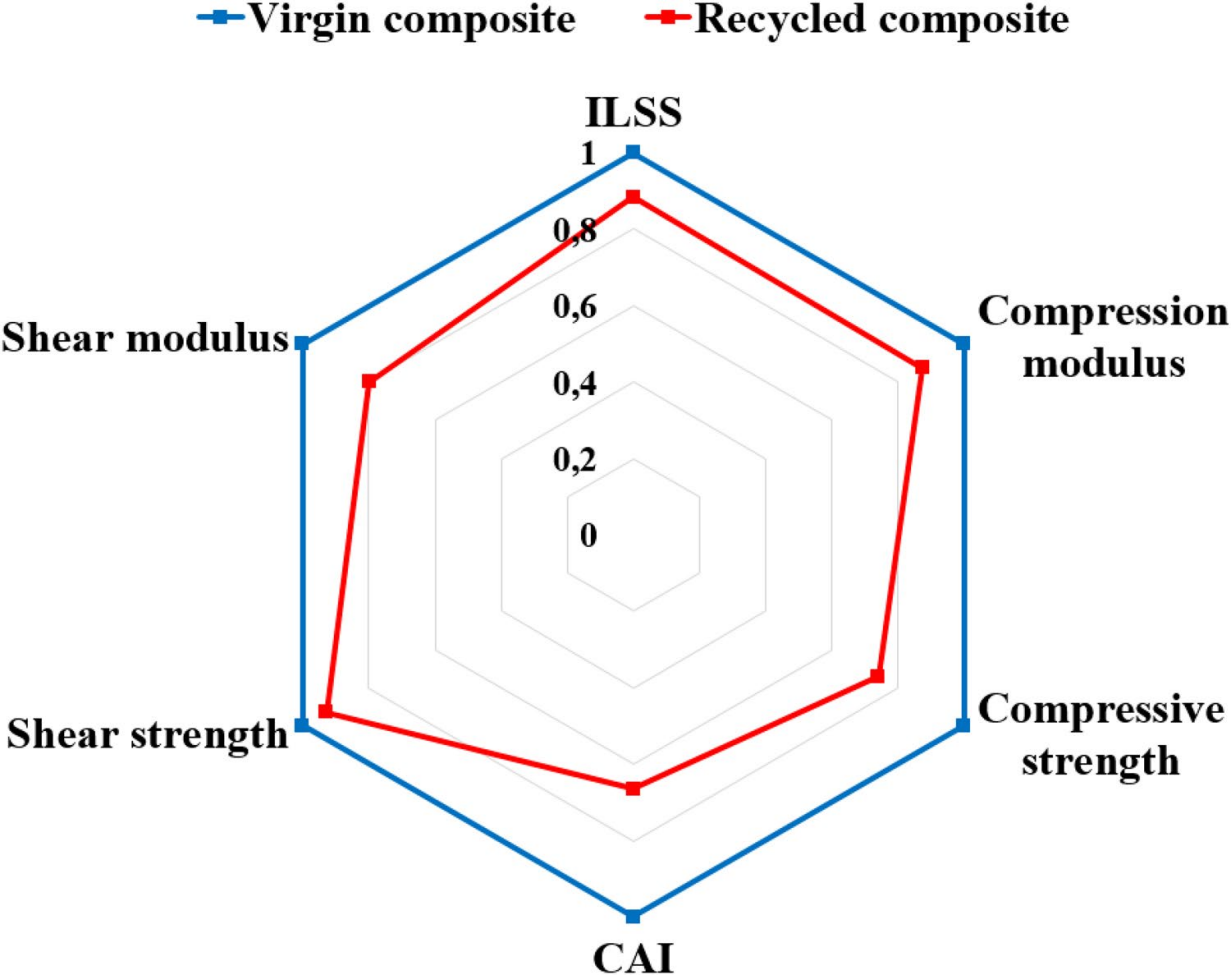
Spider chart summarizing the main mechanical propoerties for virgin and recycled composites.

A spider chart summarizing the effect of the formic acid fiber recycling on the main mechanical properties is presented in Fig. 12. The use of recycled carbon fabrics leads to a 10 to 33% decrease of the mechanical properties characterized in this work. Even though, it has not been demonstrated yet, one can anticipate that further recycling steps will not degrade more the properties. This will be confirmed in a further investigation.

**Figure 13:**
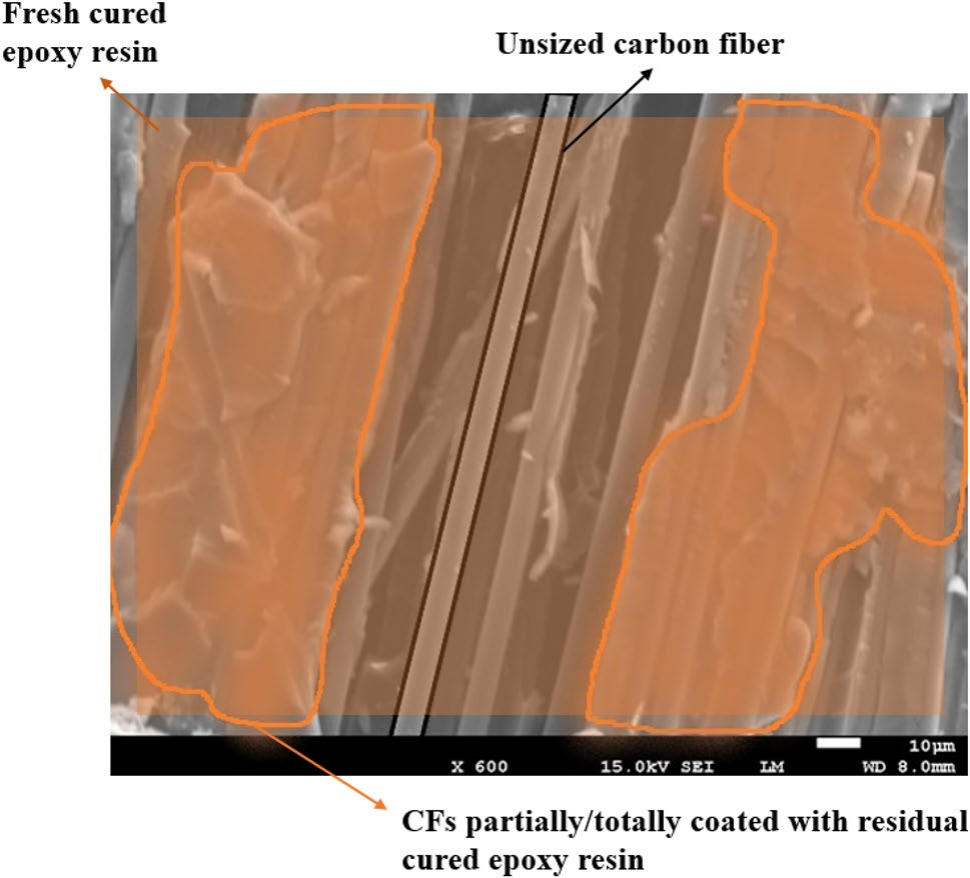
Schematic representation of the interface between fresh, residual cured epoxy and unsized carbon fibers.

**Figure 14:**
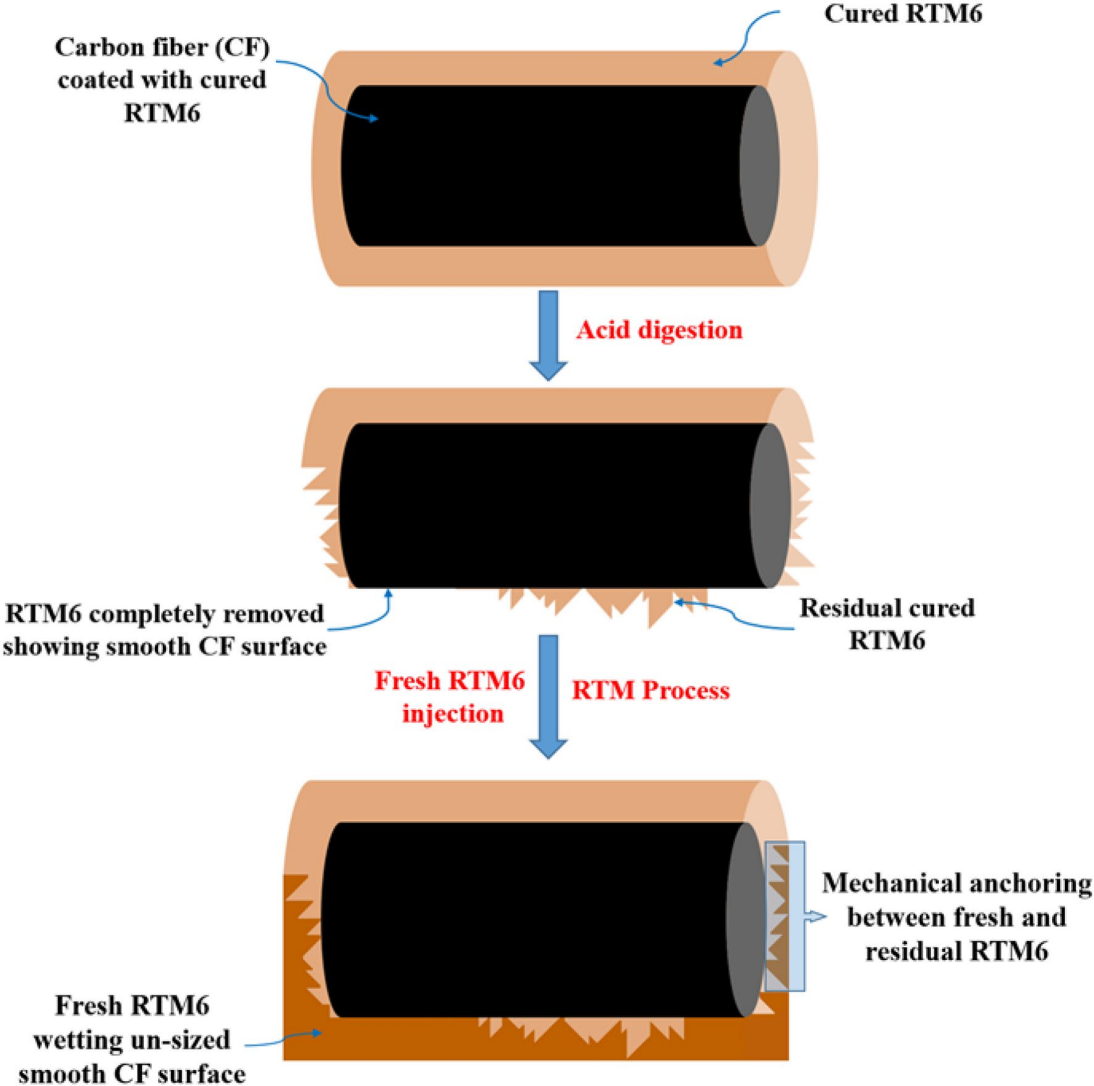
Schematic representation of the interface between fresh, residual cured epoxy and unsized carbon fibers before and after acid treatment.

**Figure 15:**
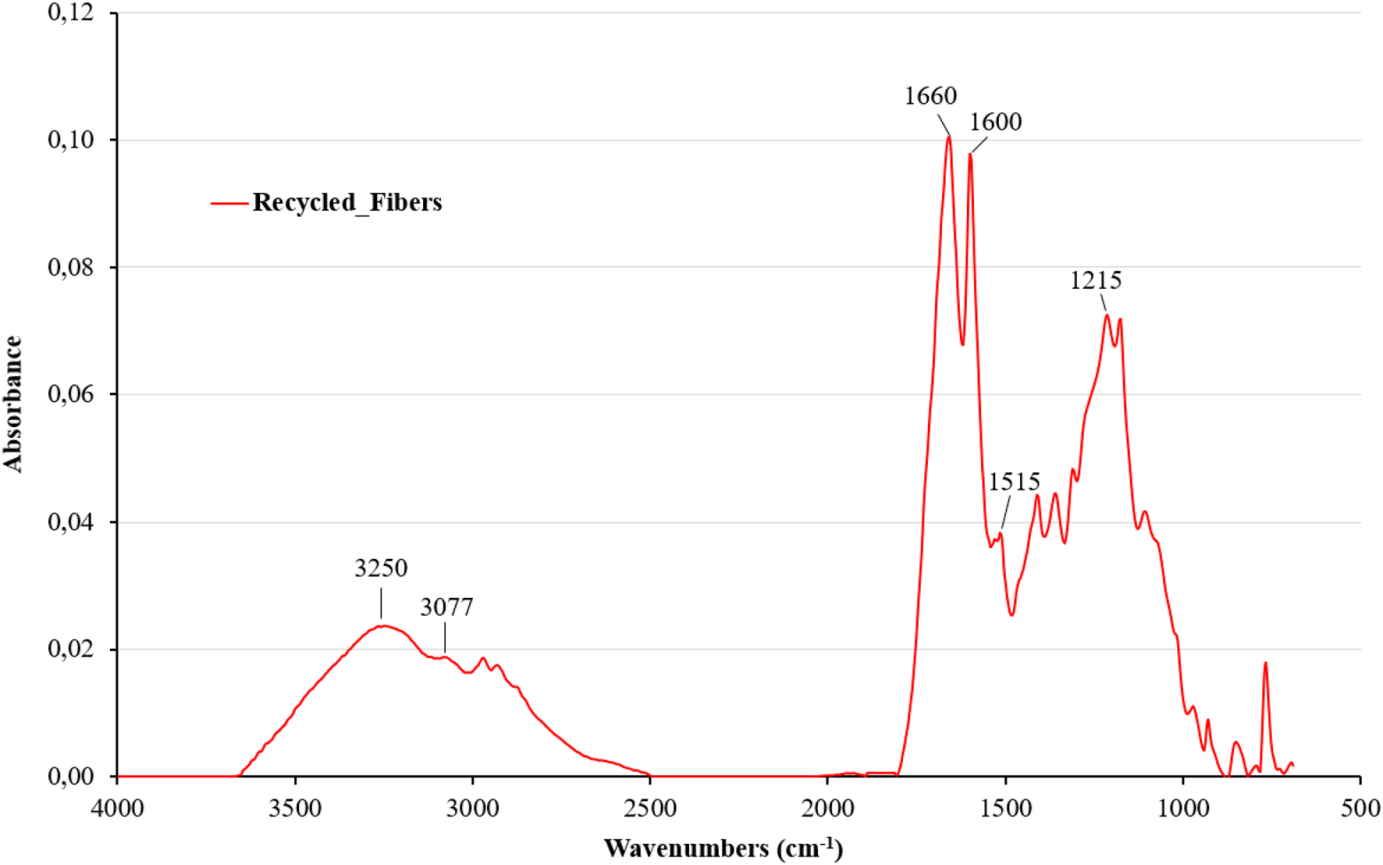
FTIR absorption spectrum of recycled carbon fibers. The absorption of virgin fibers is featureless. All absorption peaks are specific to the residual epoxy present on the surface of the recycled fibers.

Although the absence of sizing on the recycled CFs has a negative impact on strength and failure, the reduction of the mechanical properties remains limited and balanced, presumably as a result of the good cohesion between the residual cured epoxy resin and the fresh one owing to mechanical anchoring and some reactivity (epoxy groups of the fresh resin with OH groups of the residual one), despite the bad adhesion between the fresh epoxy resin and unsized carbon fibers as schematized in Figs. 13 and 14. The nanoindentation data confirm that the local behavior of the epoxy near the fibers in the recycled system is indeed very close to the one in the reference composite. Moreover, the FTIR spectrum of recycled fibers shows a number of specific vibrational bands (Fig. 15). The two bands at 3077 and 3250 cm^−1^ are assigned to N–H stretching and C = O stretching of a secondary amide, respectively. They probably result from the N-formylation of amine functions in the formic acid-treated epoxy resin as already reported by Habibi et al. and Gerack et al.^29,30^. The resulting amide groups are susceptible to react with fresh epoxy resin. The aromatic stretching band of bisphenol A is present at 1600 and 1515 cm^−1^. The peak at 1215 cm^−1^ is assigned to aromatic-hydroxyl stretching. The hydroxyl functions could also react with epoxide groups present in RTM6 fresh resin. In summary, in addition to the observed mechanical anchoring, several chemical reactions can take place between the residual RTM6 resin after formic acid attack and the freshly injected one, which both can contribute to the preservation of mechanical properties.

**Figure 16:**
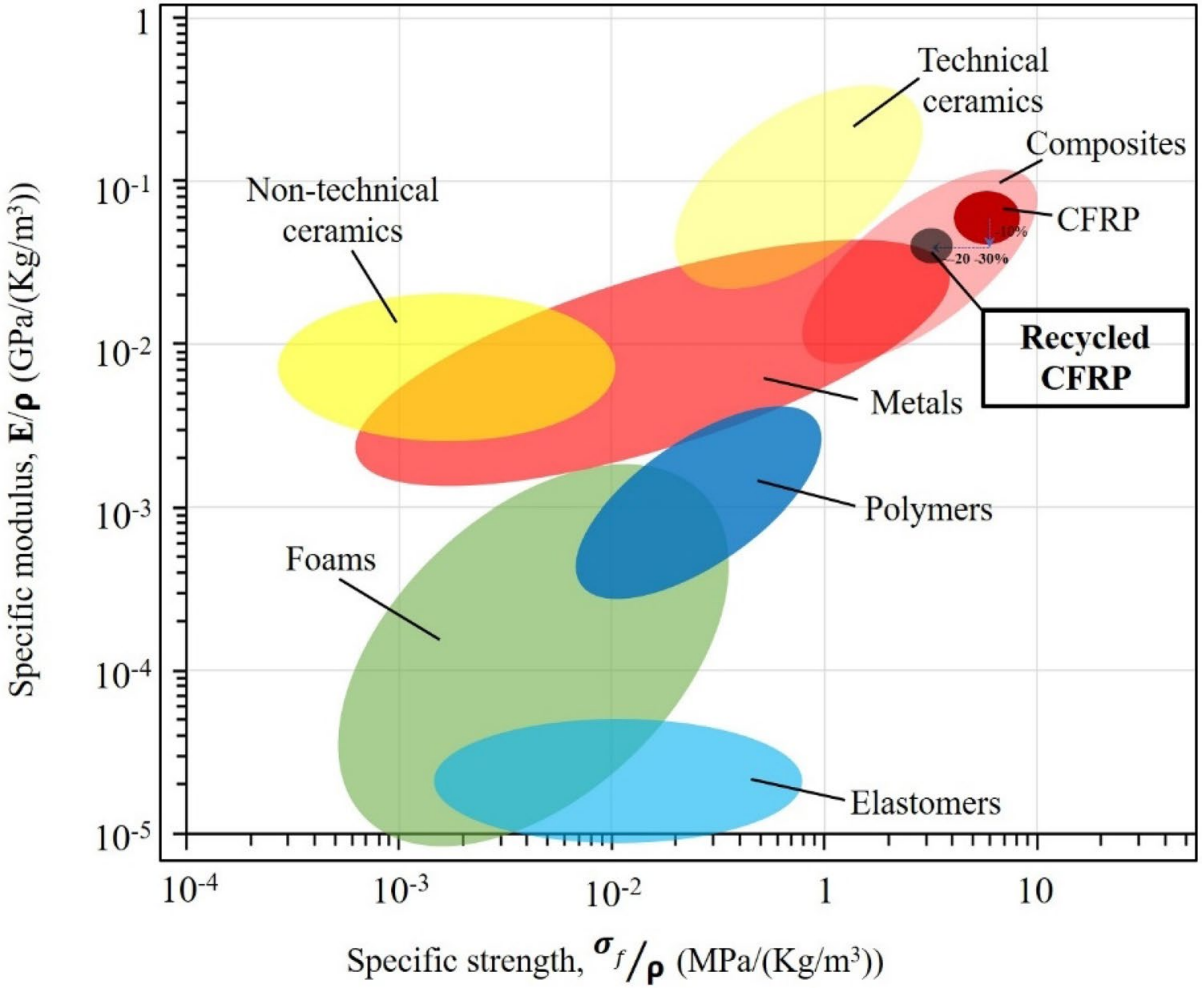
Schematic material property chart (Ashby diagram) of specific modulus versus specific strength showing the positioning of the different families of materials involving the recycled panel addressed in this study.

The composites based on reclaimed CF fabrics processed and tested in this research still maintain a very good level of performance for many applications. This is demonstrated by the schematic Ashby material property chart of Fig. 16 in terms of specific modulus (Young’s modulus normalized by density) versus specific strength (strength normalized by density) which shows the material index of the recycled composite is still above all metallic alloys. Probably, in view of the strict performance, reliability and qualification constraints demanded in aerostructures, the recycled composites would not be acceptable in aeronautics applications. However, they could find relevant application in other sectors such as in the automotive or marine fields.

## Conclusion

The potential of the recycling of CRFP composites by solvolysis process under mild conditions, with low thermal and energy inputs has been demonstrated by comparing the performance of a reference composite to a nominally identical composite made with recycled carbon fibers. The main findings of the work are the following:

- SEM and TGA results show the presence of 10 wt% residual RTM6 epoxy resin along the carbon fibers indicating that the applied conditions do not lead to a full dissolution of the cured epoxy resin.
- The digestion preferentially takes place along the edges of the composite and in the interlaminar regions due to the impact of lower carbon fibers density, favouring the acid digestion.
- The mechanical properties (ILSS, compression, CAI, Iosipescu shear and nanoindentation tests) of the recycled composites show an abatement of about 7 to 33% depending on the property when compared to the reference.
- The loss of performance is attributed to a poor adhesion between the fresh epoxy resin and the unsized carbon fibers but remain limited owing to the good interface between fresh and residual cured epoxy owing to a mechanical interlocking/anchoring effect as well as several well-identified chemical reactions.

Further recycling steps are not expected to lead to additional abatement of mechanical performance since only the interface behavior is involved. If the absence of further decrease of properties is confirmed, two options are left for the use of the recycled carbon fiber fabrics. The first option is to use them in structural applications with lower constraints on mechanical performance, keeping in mind that the lower properties still maintain these recycled composites above metallic alloys in terms of specific stiffness and strength. The second option is to introduce a re-sizing step to potentially restore their original performance.

To our knowledge, no similar studies have been reported yet. This investigation, although not yet fully optimized, is the first of its kind and paves the way for scaling up the concept to an industrial level. This recycling process preserves the virgin woven architecture and format, which can be directly used to manufacture reclaimed CFRPs. A remarkable feature is that excellent properties are attained with incomplete, and thus faster, digestion which generates a mechanical interlocking effect from resin residues left at the fiber surface. Moreover, the interest of this process is that it can treat mixed and contaminated materials with glass fiber fabrics, painted surfaces, foam cores or metal inserts, typically present in all composite structures. The process is very sustainable owing to mild room temperature/ambient pressure conditions and the possibility to recycle the acid.
